# Phenotype-oriented NGS panels for mucopolysaccharidoses: Validation and potential use in the diagnostic flowchart

**DOI:** 10.1590/1678-4685-GMB-2018-0102

**Published:** 2019-04-11

**Authors:** Ana Carolina Brusius-Facchin, Marina Siebert, Delva Leão, Diana Rojas Malaga, Gabriela Pasqualim, Franciele Trapp, Ursula Matte, Roberto Giugliani, Sandra Leistner-Segal

**Affiliations:** 1 Medical Genetics Service, Hospital de Clínicas de Porto Alegre, Porto Alegre, RS, Brazil; 2 Post-Graduation Program in Genetics and Molecular Biology, Universidade Federal do Rio Grande do Sul, Porto Alegre, RS, Brazil; 3 Molecular and Protein Analysis Unit, Centro de Pesquisa Experimental, Hospital de Clínicas de Porto Alegre, Porto Alegre, RS, Brazil; 4 Gene Therapy Center, Hospital de Clínicas de Porto Alegre, Porto Alegre, RS, Brazil; 5 Genetics Departament, Universidade Federal do Rio Grande do Sul, Porto Alegre, RS, Brazil; 6 Post-Graduation Program in Medicine, Medical Sciences, Universidade Federal do Rio Grande do Sul, Porto Alegre, RS, Brazil

**Keywords:** Lysosomal storage disease, mucopolysaccharidoses, next generation sequencing, target sequence, mutation detection

## Abstract

Mucopolysaccharidosis (MPS) are a group of rare genetic disorders caused by deficiency in the activity of specific lysosomal enzymes required for the degradation of glycosaminoglycans (GAGs). A defect in the activity of these enzymes will result in the abnormal accumulation of GAGs inside the lysosomes of most cells, inducing progressive cellular damage and multiple organ failure. DNA samples from 70 patients with biochemical diagnosis of different MPSs genotypes confirmed by Sanger sequencing were used to evaluate a Next Generation Sequencing (NGS) protocol. Eleven genes related to MPSs were divided into three different panels according to the clinical phenotype. This strategy led to the identification of several pathogenic mutations distributed across all exons of MPSs-related genes. We were able to identify 96% of all gene variants previously identified by Sanger sequencing, showing high sensitivity in detecting different types of mutations. Furthermore, new variants were not identified, representing 100% specificity of the NGS protocol. The use of this NGS approach for genotype identification in MPSs is an attractive option for diagnosis of patients. In addition, the MPS diagnosis workflow could be divided in a two-tier approach: NGS as a first-tier followed by biochemical confirmation as a second-tier.

## Introduction

Mucopolysaccharidoses (MPSs) are a group of rare genetic disorders caused by deficiency in the activity of specific lysosomal enzymes required for the degradation of glycosaminoglycans (GAGs). Thus, a defect in the activity of any of the 11 enzymes responsible for the stepwise degradation of GAGs will result in their abnormal accumulation inside the lysosomes of most cells, inducing progressive cellular damage and multiple organ failure, with consequent reduction of the quality of life and life expectancy (Neufeld and Muenzer, 2001).

MPS disorders are inherited in an autosomal recessive manner and affect males and females equally. The exception is MPS II, an X-linked recessive disorder that primarily affects males, but due to autosomal X-chromosomal translocation and non-random X-chromosome inactivation, rare female patients with MPS II have also been reported (Neufeld and Muenzer, 2001). The clinical manifestations of MPSs vary considerably, with a wide spectrum of signs and symptoms in multiple organ systems, being chronic and progressive conditions (Muenzer *et al.*, 2011).

MPS I, II, and VII are characterized by many similar clinical features that include skeletal abnormalities (dysostosis multiplex), coarse facies, corneal opacity, hearing loss, decreased pulmonary function, cardiac disease, umbilical and inguinal hernias, visceromegaly, among other problems. The disease onset, rate of progression, and manifestations vary from mild to severe. In addition to the symptomatic manifestations, patients with severe forms of MPS I, II, and VII may have cognitive impairment, typically appearing in childhood. MPS VI patients present similar somatic manifestations as MPS I, II, and VII, also with a wide spectrum of severity but with absence of cognitive impairment (Valayannopoulos *et al.*, 2005; Martin *et al.*, 2008; Muenzer *et al.*, 2009, 2011; Montaño *et al.*, 2016).

Patients with any form of MPS III present severe central nervous system degeneration with little or no somatic involvement. This disorder may be recognized by a rapid loss of social skills with aggressive behavior and hyperactivity, hirsutism, and coarse facies. The skeletal pathology is relatively mild and often becomes apparent only after a diagnosis is established (Neufeld and Muenzer, 2001; Valstar *et al.*, 2010).

Both forms of MPS IV are characterized by skeletal dysplasia, ligamentous laxity, odontoid hypoplasia, and short stature, without cognitive impairment. Patients with severe phenotype may live into their second or third decade, and those with attenuated disease may live much longer (Northover *et al.*, 1996; Tomatsu *et al.*, 2011).

MPS IX is the rarest form of MPS, with only four patients diagnosed to date, mainly with joint disease, short stature, bifid uvula, submucosal cleft palate, flat nasal bridge, generalized cutaneous swelling, multiple periarticular soft-tissue masses and popliteal cyst (Triggs-Raine *et al.*, 1999).

The purpose of this work was to validate and establish the sensitivity and specificity of NGS panels to identify genetic mutations in MPS patients previously diagnosed and genotyped. Panels were designed to be phenotype-oriented, considering the feasibility of this approach as a first-tier alternative followed by biochemical confirmation as a second-tier.

## Subjects and Methods

### Patients

Samples from 70 MPS patients (8 MPS I, 12 MPS II, 23 MPS III, 17 MPS IV, 6 MPS VI, and 4 MPS VII) and eight controls were included in this study. All patients had a previous biochemical diagnosis and were already genotyped by Sanger sequencing. The study was approved by the Hospital de Clinicas de Porto Alegre Research Ethics Committee, which is recognized by the Office for Human Research Protections as an Institutional Review Board (IRB0000921).

### AmpliSeq gene panels

Ion Torrent semiconductor technology is able to load onto an Ion 314^TM^, 316^TM^ and 318^TM^ chip, 10 Mb, 100 Mb and 1 Gb of sequence per run, respectively. Targeted NGS can be achieved by Ion AmpliSeq technology (Thermo Fisher Scientific), an ultrahigh-multiplex PCR amplification strategy that uses very low input genomic DNA for a simple and fast library construction of specific human genes or genomic regions (Buermans and den Dunnen, 2014). To set up a fast and comprehensive assay for molecular analysis of MPS based in NGS sequencing using the Ion Torrent Personal Genome Machine^TM^, we designed three customized AmpliSeq^TM^ panels for sequencing eleven genes (*IDUA, IDS, SGSH, NAGLU, HGSNAT, GNS, GALNS, GLB1*, *ARSB*, *GUSB*, and *HYAL1*) related with MPSs. These panels were validated in 78 samples (70 MPS patients and 8 controls) previously sequenced by Sanger method.

The 11 genes related with MPS disease were divided into three different customized panels according to the similarity of clinical presentations. Panel 1, first comprised MPS types I, II, VI and VII and was later redesigned to include MPS IX; Panel 2 included all MPS III types, and Panel 3 included the MPS IV types A and B. AmpliSeq^TM^ primer panels were designed by Ion AmpliSeq Designer software (Thermo Fisher Scientific) resulting in a two-pool design for each panel.

### DNA isolation

Genomic DNA (gDNA) was extracted from fresh peripheral blood samples using a salting out method or automated extraction using iPrep Purelink gDNA Blood kit (Invitrogen). DNA sample quantity and purity of the nucleic acid samples were assessed using a Nanodrop 1000 (Thermo Fisher Scientific) specrophotometer. All of the gDNAs had 260/280-nm absorbance ratios between 1.8 and 2.0. Patient DNA samples were adjusted to a final concentration of 10 ng/μL.

### Library preparation

The customized Ion AmpliSeq^TM^ panel was processed using the Ion AmpliSeq Library Kit 2.0 (Thermo Fisher Scientific) according to the manufacturer’s recommendations, starting from 10 ng of gDNA per pool. The samples were barcoded with the Ion Express Barcode Kit (Thermo Fisher Scientific) to optimize patient pooling on the same sequencing chip. The number of samples by chip was determined taking into account the total length of each panel, aiming for at least 100X coverage (number of reads per amplicon).

### Ion Torrent PGM sequencing

Template preparation was performed using an Ion One Touch 2 System (Thermo Fisher Scientific) and an Ion One Touch ES (Thermo Fisher Scientific) following the latest version of the manufacturer’s manuals. The template-positive Ion Sphere Particles (ISP+) were sequenced on an Ion Torrent Personal Genome Machine (PGM) (Thermo Fisher Scientific) using the 314 Chip v2, following the Ion PGM Sequencing 200 Kit v2 manual. We used 500-flow runs, which support a template read length of approximately 200 bp.

### Bioinformatics analyses

The raw data was processed with the Torrent Suite Software v5.0 (Thermo Fisher Scientific) using the standard pipeline parameters. Read alignment and variant identification was done with the Torrent Mapping Program (TMAP) v3.4.1 and Torrent Variant Caller (TVC) v5.0 software. We used hg19 (Human Genome version 19, UCSC) as reference for alignment with TMAP, and a BED (Browser Extensible Display, UCSC) file to define our regions of interest on TVC (Merriman *et al.*, 2012). The Integrative Genome Viewer (IGV; Broad Institute) was used for the analysis of depth coverage, sequence quality, and variant visualization (Thorvaldsdóttir *et al.*, 2013).

The Coverage Analysis plugin from Torrent Suite software was also used to establish four different types of coverage charts. For each sample, the amplicon coverage summary file was downloaded, sorted according to the .bed reference file, and the total reads information was used to create a unique spreadsheet for all samples of a given panel. Depth values of each amplicon were divided by the median depth of the batch, following by calculation of the log_25_ ratio to access comparisons between sequenced samples. To evaluate the intra-run performance of each amplicon, the mean of log_25_ value and the standard deviation were calculated. In order to analyze the inter-run reproducibility, we concatenated the intra-run results. We also used the normalized total reads mean values to explore the depth of each sequenced gene.

## Results

In this study, we aimed to evaluate the sensitivity and specificity of three custom Ampliseq panels designed for amplifying the coding regions of MPS related genes. These designed panels allowed the analysis of 81 out of 90 exons, targeting 90-95% of the genes (Table 1a,b). Libraries of 78 samples, previously sequenced by Sanger sequencing, were re-sequenced by NGS in 12 separated runs: six for Panel 1 and three for Panels 2 and 3. To ensure adequate depth and coverage for variant identification, we sequenced 8-16 barcoded samples on a 314 chip. For this sample set, we obtained an average of mapped reads of 76,000 for Panel 1, 58,000 for Panel 2, and 47,000 for Panel 3; the average of reads on target, depth of coverage, and uniformity varied from gene to gene (Table 1c).

**Table 1:** Coverage metrics of gene panels.

a) Panel metrics			
	Size	Number of amplicons	Missed bp	Coverage (%)
Panel 1	13.42Kb	83	342	95.9
Panel 2	7.81Kb	66	435	95.1
Panel 3	8.61Kb	49	562	90.1

b) Target genes coverage and missed regions			
Gene	Target size (bp)	Missed (bp)	Covered (%)
IDUA	2,256	209	90.74
IDS	2,079	0	100
ARSB	1,820	96	94.73
GUSB	2,208	37	98.32
SGSH	1,597	0	100
NAGLU	2,298	294	87.21
HGSNAT	2,106	141	93.3
GNS	1,813	0	100
GALNS	2,657	541	79.64
GLB1	3,049	21	99.31

c) Panel coverage metrics						
		Number of samples	Mapped reads	Reads on target	Depth of coverage	Uniformity of coverage
Panel 1						
	*IDUA*	8	77,000	79%	1160	88%
	IDS	12	76,000	81%	641	87%
	ARSB	6	75,000	82%	841	86%
	GUSB	4	76,000	82%	580	86%
Panel 2						
	SGSH	11	56,000	94%	693	90%
	NAGLU	8	61,000	94%	764	89%
	HGSNAT	2	58,000	76%	477	91%
	GNS	2	-	-	-	-
Panel 3						
	*GALNS*	13	42,000	67%	565	87%
	*GLB1*	4	53,000	83%	800	87%

We were able to identify 250 variants in the 70 samples. Variants were filtered according to: a) minimum coverage of 100X; b) variant detection in both strands; and c) variant frequency < 1%. After filtration, 65 variants remained, including 48 missense/nonsense mutations and 17 pathogenic indels. Overall, we were able to identify 98% of all variants previously identified by Sanger method, 96% in Panel 1, 100% in Panel 2, and 100% in Panel 3, showing a high sensitivity in detecting different types of mutations using this approach (Table 2). Furthermore, no other known pathogenic mutation was detected, in addition to those found by Sanger sequencing, showing 100% specificity of our assays.

**Table 2:** Number of variants identified by Sanger and NGS.

	Gene	Pathogenic point mutations		Pathogenic indels		Other variants^*^
Panel 1		Sanger	NGS	Sanger	NGS	
	*IDUA*	6	3	0	0	44
	*IDS*	6	6	6	6	10
	*ARSB*	4	4	2	2	25
	*GUSB*	4	4	1	1	18
Panel 2						
	*SGSH*	6	6	2	2	21
	*NAGLU*	9	9	3	3	19
	*GNS*	0	0	0	0	14
	*HGSNAT*	2	2	0	0	11
						
						
Painel 3	*GALNS*	12	12	1	1	15
	*GLB1*	2	2	2	2	8

* Non-pathogenic mutations: synonymous or polymorphisms

**Figure 1:**
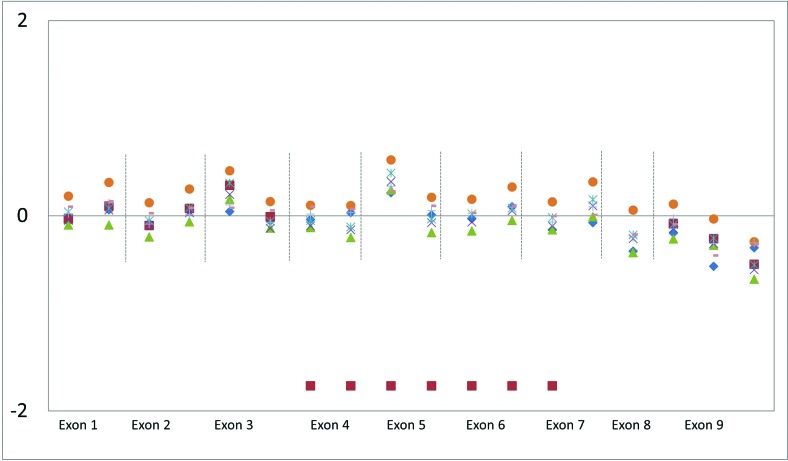
Deletion visualized in the decrease coverage of log_25_ values. Relative copy number derived from coverage data per target. The red squares mark the probable deletion of the exons IV to VIII of *IDS* gene.

A partial deletion present in two MPS II patients could be inferred from coverage graphs. These events are noticed as decreased coverage of consecutives exons, compared with the median observed for samples sequenced in the same batch (Figure 1). Nevertheless, the exact breakpoints could not be determined, and other technologies, such as array-CGH, should be used for this purpose.

Exon 1 of some genes (*IDUA*, *ARSB*, *NAGLU*, *HGSNAT*, and *GALNS)* and other four exons (exon 10 of *IDUA*, exon 7 and 11 of *GUSB,* exon 5 of *GLB1*), failed in the design of amplicons, probably due to high GC content and/or presence of repeat regions.

**Figure 2:**
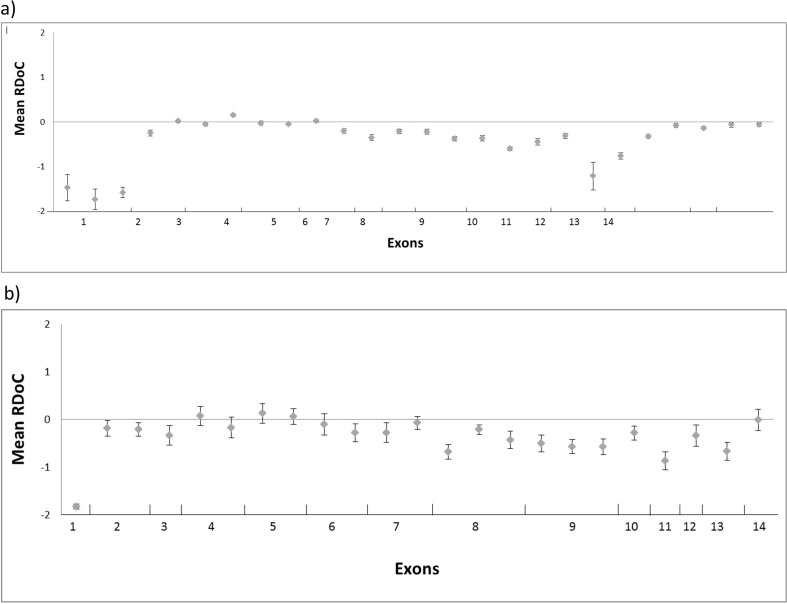
*IDUA* gene amplicon coverage. (a) Intra-run relative depth of coverage (RDoC) at 14 exons corresponding to 8 samples sequenced in 6 runs; (b) *IDUA* gene amplicon coverage of second design. Intra-run RDoC at 14 exons corresponding to 8 samples sequenced in 1 run.

The *IDUA* gene had low amplification efficiency of some amplicons, as can be seen in Figure 2a, which shows the log_25_ ratio for eight samples sequenced in different runs. Consistently, all of them fell below the median depth of the batch in most of exons; as MPS I was the third-most frequent MPS in our routine analysis, we decided to re-design Panel 1, improving exon coverage.

A run with eight samples was performed in order to evaluate the new panel design for the *IDUA* gene. We could identify 12 out of 14 mutations previously detected by Sanger sequencing. The other two mutations not detected were in exon 1, and no amplicons were designed by Ampliseq^TM^ design software for this region. The median depth of the batch showed a high coverage depth when compared with the first panel designed (Figure 2b). Calling of variants in highly homologous regions that cannot be accurately detected by NGS can often be solved by other methods, such as Sanger sequencing, through the design of specific primers, or by other methods, such as bioinformatics, through realignment.

**Figure 3:**
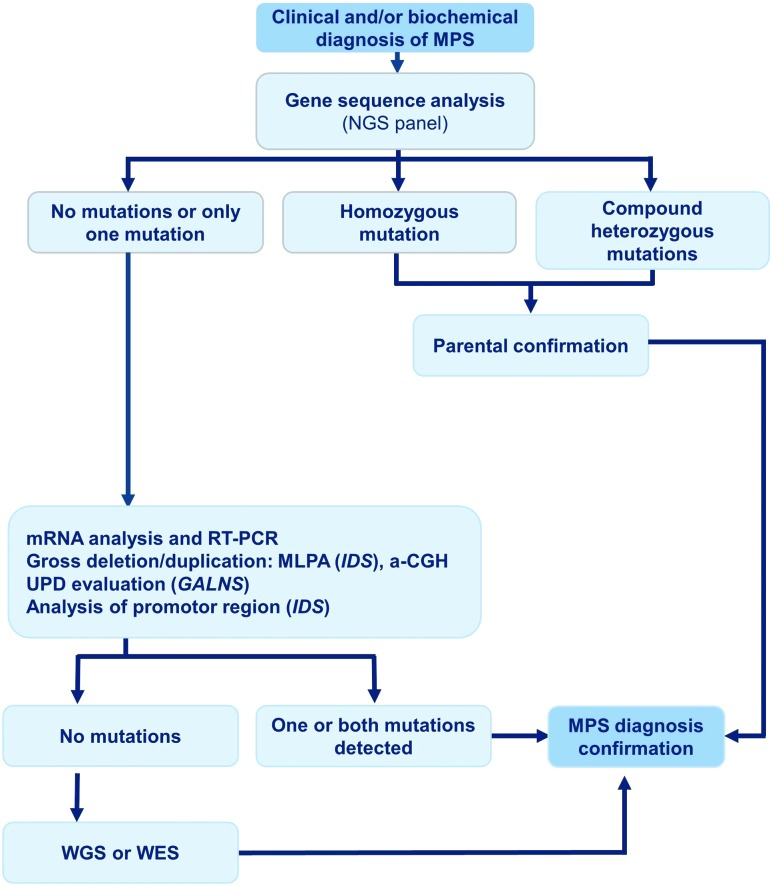
Flow Chart for MPS investigation.

The *HYAL1* gene, which is associated with MPS IX, was included in the re-designed panel in order to have a complete coverage of the MPSs genes. This analysis was validated using only the eight normal samples, once this type of MPS is very rare and no positive case was available. The analysis showed a great coverage and depth for all exons. Although the finding of a specific enzyme deficiency in leucocytes or fibroblasts confirms the diagnosis, molecular analysis of the respective gene is recommended, whenever possible (Figure 3).

## Discussion

MPSs are genetic metabolic diseases that can be caused by mutations in several distinct genes, each one coding for a specific enzyme in the GAG degradation pathway. Symptoms may be similar in some MPS types, and nowadays definitive diagnosis is achieved by enzyme assays, usually confirmed by genotype analysis.

Once a clinical suspicion of MPS is raised, biochemical studies are usually performed with diagnostic purposes. The first step in this process aims to identify if the levels of total GAGs in urine are increased and which are the GAGs species excreted in urine. Quantitation of urinary GAGs is usually colorimetric (de Jong *et al.*, 1989), and the qualitative pattern can be obtained by thin-layer chromatography (TLC), electrophoresis, or tandem mass spectrometry (TMS) techniques (Hopwood and Harrison, 1982; Dembure *et al.*, 1990; Auray-Blais *et al.*, 2012; Tomatsu *et al.*, 2014). The gold standard diagnosis of MPS is based on determination of the enzyme activity in plasma, leukocytes, or fibroblasts by fluorometric methods (Voznyi *et al.*, 2001). Dried blood spots (DBS) could also be used for most enzyme assays, but it is usually recommended to confirm positive results in leukocytes or fibroblasts. These biochemical tests are often laborious and require specialized personnel and specific substrates. Sometimes, it is hard to obtain viable samples, especially when there is the need to travel long distances and across international borders.

Although the biochemical tests (enzyme assays and GAG analysis) are sufficient to confirm the diagnosis of MPS, genetic analysis of the specific genes involved is necessary for the characterization of the molecular defect and prognosis prediction. This enables the detection of carrier status of relatives (especially important for female relatives of MPS II patients) and accurate genetic counseling. The knowledge of the causative mutation also allows a faster and more precise prenatal diagnosis in future pregnancies in affected families.

Over the past 30 years, Sanger sequencing technology has been regarded as the gold standard to identify sequence alterations in the target region, being an accurate approach for molecular diagnosis. This method relies on analyzing individual genes, usually exon-by-exon, being expensive and time consuming (Sanger *et al.*, 1977).

In this sense, we developed a custom panel for molecular analysis of MPS patients using NGS, thus allowing the simultaneous sequence of multiple genes grouped according to the MPS phenotype. In our study, we chose to group MPS patients according to their clinical symptoms instead of developing a comprehensive panel for all disease types. This allows maximizing the number of patients per run, avoiding unnecessary sequencing of genes that do not fit the clinical hypothesis.

The use of the NGS mutation detection method compared with Sanger sequencing led to the identification of 250 variants and 90% coverage of the 11 genes involved in MPSs etiology. The high sensitivity of our assay could be demonstrated in the detection of 94% mutations, including missense, nonsense, splice sites, in-frame deletions, and large deletions, such as a deletion of four exons in the *IDS* gene.

On the other hand, in five samples, the genotype could not be defined. This was due to mutations located in regions not covered or with low amplification (< 100X), caused by a high GC content or repeat regions. For these regions, for which Ion Torrent PGM was unable to sequence, or for regions not designed by Ampliseq design software, other methodologies, such as Sanger sequencing must be used for a complete gene amplification.

Moreover, due to the inability of NGS to adequately cover some GC-rich regions, as seen for the *IDUA* gene (MPS I), we re-designed Panel 1, changing primers for these regions in order to improve read depth, because there are pathogenic mutations previously reported in these regions. We did not redesign the other panels that presented genes without coverage or with low coverage depth due to the presence of high GC content in the segments.

Other studies had used NGS to diagnose lysosomal storage disorders by whole-exome approaches (Fernández-Marmiesse *et al.*, 2014; Prada *et al.*, 2014). Selmer *et al.* (2012) described a mild form of MPS IIIB and illustrated the diagnostic potential of targeted NGS in Mendelian disease with unknown etiology. Wei *et al.* (2011), reported a novel disease causing mutation in the *IDS* gene using this strategy.

NGS is limited by the enormous amounts of genomic data generated after sequencing of large genomic regions, making interpretation a big challenge. We were able to minimize this by our targeted sequencing strategy. Assigning pathogenicity for previously undetected variants can also be difficult (Grada and Weinbrecht, 2013; Barba *et al.*, 2014). In the specific scenario, the existence of validated biochemical tests can help to establish which alterations are disease-causing. However, if more than two variants of unknown significance are found in the same gene (or one in the case of X-linked *IDS*) there might remain the doubt of which one (if any) is a non-pathogenic variant.

Additionally, complex rearrangements and large gene deletions could not be detected by the Ion Torrent platform, requiring the use of other technologies, including aCGH, MLP, and cDNA analysis by Sanger sequencing, to complement the NGS molecular analysis.

In conclusion, the Ion Torrent PGM was found to be a fast tool with a high capacity of sequencing, allowing mutation detection of multiple genes and patients at the same time, improving the molecular diagnosis approach. This massive parallel sequencing technology allows the sequencing of large genomic regions in a short time and at a low cost. In addition, NGS platforms are becoming more popular and consequently more affordable to smaller centers. Thus, we suggest this NGS protocol as a first-tier for MPS diagnosis followed by a second-tier biochemical confirmation.
